# Effect of Alcoholism upon Paternity

**Published:** 1898-09

**Authors:** 


### Effect of Alcoholism Upon Paternity.

Anthony (*Gazette hebdomadaire de medecine et de chirurgie*) records the case of a woman of seventeen who married an intemperate husband, by whom she had five weakly children, of which four died within ten days of birth, and one succumbed before reaching four years. The mother then married again, to a perfectly robust man, by whom she has had two children, both stout and healthy.—*Philadelphia Polyclinic.*

